# Improved Microbial Community Characterization of 16S rRNA via Metagenome Hybridization Capture Enrichment

**DOI:** 10.3389/fmicb.2021.644662

**Published:** 2021-04-27

**Authors:** Megan S. Beaudry, Jincheng Wang, Troy J. Kieran, Jesse Thomas, Natalia J. Bayona-Vásquez, Bei Gao, Alison Devault, Brian Brunelle, Kun Lu, Jia-Sheng Wang, Olin E. Rhodes, Travis C. Glenn

**Affiliations:** ^1^Department of Environmental Health Science, University of Georgia, Athens, GA, United States; ^2^Interdisciplinary Toxicology Program, University of Georgia, Athens, GA, United States; ^3^Savannah River Ecology Laboratory, University of Georgia, Aiken, SC, United States; ^4^Institute of Bioinformatics, University of Georgia, Athens, GA, United States; ^5^Daicel Arbor Biosciences, Ann Arbor, MI, United States

**Keywords:** amplicon, microbial diversity, microbiome, mock communities, next generation sequencing, shotgun libraries, target enrichment

## Abstract

Environmental microbial diversity is often investigated from a molecular perspective using 16S ribosomal RNA (rRNA) gene amplicons and shotgun metagenomics. While amplicon methods are fast, low-cost, and have curated reference databases, they can suffer from amplification bias and are limited in genomic scope. In contrast, shotgun metagenomic methods sample more genomic regions with fewer sequence acquisition biases, but are much more expensive (even with moderate sequencing depth) and computationally challenging. Here, we develop a set of 16S rRNA sequence capture baits that offer a potential middle ground with the advantages from both approaches for investigating microbial communities. These baits cover the diversity of all 16S rRNA sequences available in the Greengenes (v. 13.5) database, with no sequence having <78% sequence identity to at least one bait for all segments of 16S. The use of our baits provide comparable results to 16S amplicon libraries and shotgun metagenomic libraries when assigning taxonomic units from 16S sequences within the metagenomic reads. We demonstrate that 16S rRNA capture baits can be used on a range of microbial samples (i.e., mock communities and rodent fecal samples) to increase the proportion of 16S rRNA sequences (average > 400-fold) and decrease analysis time to obtain consistent community assessments. Furthermore, our study reveals that bioinformatic methods used to analyze sequencing data may have a greater influence on estimates of community composition than library preparation method used, likely due in part to the extent and curation of the reference databases considered. Thus, enriching existing aliquots of shotgun metagenomic libraries and obtaining modest numbers of reads from them offers an efficient orthogonal method for assessment of bacterial community composition.

## Introduction

The study of microbes is critically important, as they have many essential roles in ecosystem function, disease pathology, host physiology, and possibly assessing infectious disease outbreaks (Dueker et al., 2018; Gallardo-Escárate et al., 2020). As microbial communities can often be highly diverse and complex, it can be challenging to identify rare taxa in complex environmental samples (e.g., soil, freshwater, etc.) with traditional and modern techniques (i.e., culturing, 16S amplicons, or metagenomic shotgun libraries). Advances in sequencing technologies have transformed traditional microbiology. Microbial communities that were previously considered indiscernible or unstudied, can now be investigated at greater depths than ever before from many different environmental systems (Gilmour et al., 2010; Kustin et al., 2019).

For decades, the 16S small subunit ribosomal RNA (rRNA) gene has been the gold standard marker for microbial molecular taxonomic research (Woese and Fox, 1977; Meola et al., 2015), as this highly conserved gene contains nine rapidly evolving hypervariable regions that aid in species identification (Yuan et al., 2015). Amplicon sequencing, targeting the 16S rRNA, is a cost-effective and high-throughput method used to study aquatic, terrestrial, food- and host-associated microbial communities (Logares et al., 2014; Polka et al., 2015; Jiang et al., 2016; Jousselin et al., 2016; Jouglin et al., 2019; Suenami et al., 2019; Ziegler et al., 2019). However, studies relying on 16S rRNA amplicon sequencing have limitations and biases. Relevant biases in 16S rRNA amplicon sequencing are associated with DNA extraction, amplification via PCR, sequencing, and sequence analysis (Kennedy et al., 2014; Knight et al., 2018). Specifically, PCR biases include primer bias (Klindworth et al., 2013; Kelly et al., 2019) and varying GC content (Aird et al., 2011). Additional limitations associated with amplicon sequencing include challenges in the taxonomic characterization of microbial communities, as well as accuracy and availability of reference databases (Kennedy et al., 2014; Poretsky et al., 2014; Ritari et al., 2015; Knight et al., 2018). Furthermore, the selection of the hypervariable region used for the amplicon analysis (i.e., V1–V3; V3–V4; V4; etc.) can lead to differences in bacterial identification (Vetrovsky and Baldrian, 2013; Martinez-Porchas et al., 2016).

In more recent years, metagenomic shotgun sequencing has aimed to characterize taxonomic profiles of unique clade-specific marker genes to provide a balanced view of community composition and function (Neelakanta and Sultana, 2013; Knight et al., 2018). However, metagenomic sequencing has its own limitations; genomic DNA may contain non-target DNA (e.g., human DNA), which can affect downstream analysis (e.g., mis-assemblies of sequence contigs, spurious reads) thus leading to inaccurate conclusions (Schmieder and Edwards, 2011; Gasc and Peyret, 2018). Also, metagenomic libraries are more expensive, take longer to prepare, and are much more complex than amplicon libraries, requiring more computational effort (Sekse et al., 2017). In particular, it is difficult to identify low abundance genetic traits and rare taxa using metagenomic libraries, and extensive deep sequencing is often required to do so (Lasa et al., 2019). In summary, shotgun sequencing is less biased and yields data on many genomic regions, but the main tradeoffs are higher costs of library preparation, sequencing, analysis, and potential for differences vs. 16S amplicons (see below).

Mock communities can be used to help establish ground truth in microbial diversity studies, in particular when comparing different library preparation methods (Costea et al., 2017; Rausch et al., 2019). Rausch et al., 2019 provided a comparison of 16S rRNA amplicon sequencing and metagenomic sequencing, and revealed similar community makeup (i.e., abundance and taxa diversity) of their shallow mock community regardless of library type. Conversely, other studies have found key differences in abundance and taxa of mock communites attributed to wet-laboratory methods (Costea et al., 2017; Rausch et al., 2019). However, some of these differences may be attributed to varying bioinformatic tactics.

In terms of bioinformatic analyses, advantages and limitation of methods, reference databases, and software have been vastly described for both 16S rRNA and metagenomic strategies (Truong et al., 2015; Callahan et al., 2016; Costea et al., 2017; Escobar-Zepeda et al., 2018; Rausch et al., 2019). The variation among these can lead to a lack of sensitivity and specificity that may contribute to wrong classifications and/or no classification at a specific taxonomic level, and erroneous abundance assignments (Escobar-Zepeda et al., 2018). In particular, it can be challenging to analyze environmental samples, as most reference databases are based on human commensals (Dueholm et al., 2020). Furthermore, the number of 16S rRNA gene copies can vary widely between bacterial species, and may contribute to biases in abundance estimates (Vetrovsky and Baldrian, 2013).

Both strategies (i.e., 16S rRNA amplicon and metagenomic shotgun libraries) present their own challenges and variations in analyses (Knight et al., 2018), but metagenomic shotgun libraries tend to perform at a higher sensitivity and specificity than 16S rRNA amplicon data (Escobar-Zepeda et al., 2018). For metagenomic data, programs like MetaPhlAn2 may be used to classify and estimate the relative abundance of microbial cells by mapping reads against marker sequences to classify the sequences at the sub-species to higher taxonomic levels (i.e., marker-gene approach) (Segata et al., 2012; Truong et al., 2015). Whereas 16S rRNA amplicon data is commonly analyzed by inferring representative sequences using a variety of methods, some of which are influenced by fragment size and 16S region (Edgar, 2013; Callahan et al., 2016). Furthermore, some methods used to assign operational taxonomic units may result in limited resolution at lower taxonomic levels (e.g., genus and species levels), as even organisms that share 98.75% sequences may be different species (Mysara et al., 2017). Reference databases for 16S rRNA are much more extensive than those for metagenomic analyses, which is key for superior analysis, particularly in samples that are not from human commensals (Escobar-Zepeda et al., 2018). However, variation in taxonomic classification and abundance has also been associated with the use of different reference databases (Jovel et al., 2016; Rausch et al., 2019).

Hybridization capture (also known as sequence capture, target capture, or targeted sequence capture) is an enrichment technique that uses a set of biotinylated DNA or RNA baits that are complementary to DNA sequences of interest to increase the proportion of DNA fragments of interest within DNA libraries, subsequently characterizing the DNA by massively parallel sequencing (Lasa et al., 2019). Hybridization capture assays have been designed previously for the 16S rRNA gene, using 15–1,402 baits (Gasc and Peyret, 2018; Barrett et al., 2020). Additional hybridization capture bait sets have been designed for a variety of microbial projects, such as sets of defined pathogens or particular genes, including virulence genes for *Vibrio* spp. that infect oysters (Lasa et al., 2019), bifidobacterial in the gut of mammals (Lugli et al., 2019), and antibiotic resistance genes (Guitor et al., 2019). Importantly, unlike other culture independent techniques, hybridization capture provides greater phylogenetic resolution and increased sensitivity, while requiring fewer sequencing reads (Lasa et al., 2019; Barrett et al., 2020). More specifically, 16S rRNA capture baits provide a cost-effective way to identify bacteria in diverse environmental samples and identify rare taxa.

Here, we present a hybridization capture method (i.e., 16S-cap) to enrich metagenomic shotgun libraries for DNA sequences of 16S rRNA genes. Our protocol improves on the existing methods by including many more baits that better cover known sequence variation in 16S databases, taking advantage of the extensive reference databases and ease of analyses of 16S rRNA sequences for taxonomic classification and decreasing bias introduced from primer affinity, while reducing sequencing costs per sample compared to unenriched metagenomic libraries. For microbes, targeted sequence capture techniques for 16S rRNA have shown more accurate representation of microbial communities compared to traditional methods (i.e., 16S rRNA amplicons, shotgun libraries) (Gasc and Peyret, 2018). We provide a comparison of traditional methods for assessing composition of microbial communities (i.e., 16S rRNA amplicons and metagenomic shotgun libraries) with our 16S-cap method to characterize *in silico* mock, *in vitro* mock, and real microbial communities from genomic data.

## Materials and Methods

### Samples and DNA Extraction

We used two commercial standard genomic DNA mock community collections to characterize simple communities (HM-276D, BEI Resources, Manassas, VA; D6306, Zymo Research, Irvine, CA). For complex communities, we used a subset of fecal samples from previous studies that examined the impacts of environmental xenobiotic agents on the gut microbial communities of rodent models (Gao et al., 2017; Wang et al., 2018). The first study examined carbamate insecticide in male C57BL/6 mice (i.e., *Mus musculus*) (Gao et al., 2017), and the second examined green tea polyphenols in female Sprague-Dawley rats (i.e., *Rattus norvegicus*) (Wang et al., 2018). DNA was extracted using Qiagen Fast DNA Stool Mini Kit (QIAGEN, Valencia, CA, United States) or PowerSoil DNA Isolation Kit (Mo Bio Laboratories, Carlsbad, CA, United States). Details on experimental design and extractions are previously described (Gao et al., 2017; Wang et al., 2018).

### 16S rRNA Amplicon Metabarcoding

The primer pairs targeting the V3 and V4 16S regions (S-D- Bact-0341-b-S-17 and S-D-Bact-0785-a-A-21) (Klindworth et al., 2013) were used for amplification of the 16S rRNA gene in rat fecal samples and mock communities; and the primer pair targeting the V4 region (515-F and 806-R) (Caporaso et al., 2012) was used on the mouse fecal samples. We created indexed fusion primers with TruSeq compatible sequencing oligos as previously described using the *Adapterama I* and *Adapterama II* systems (Glenn et al., 2019a, b) to generate amplicon libraries using two rounds of PCR [Method 5 of Table 3 from Glenn et al. (2019b)]. For the first PCR, we prepared individual 25 μL PCR reactions for each sample using KAPA HiFi reagents (KAPA Biosystems, Wilmington, MA, United States). Each PCR reaction mix included 5 μL 5× KAPA HiFi buffer, 0.75 μL 10 mM dNTPs, 0.5 μL KAPA HiFi HotStart, 1.5 μL 5 μM forward indexed-fusion primer, 1.5 μL 5 μM reverse indexed-fusion primer, and 1 μL of 20 ng/μL DNA. PCR conditions were as follows: initial denaturation at 95°C for 3 min; 15–18 cycles of 95°C for 20 s, 60°C for 30 s, and 72°C for 30 s; final extension at 72°C for 5 min.

In preparation for the second PCR, we normalized individually indexed PCR products with a SequalPrep Normalization Plate Kit (Invitrogen, Carlsbad, CA, United States) according to manufacturer’s protocols or by pooling them together based on agarose gel band brightness. These pools served as the template for a second limited cycle PCR. Each 25 μL PCR reaction mix included: 5 μL 5× KAPA HiFi buffer, 0.75 μL 10 mM dNTPs, 0.5 μL KAPA HiFi HotStart, 2.5 μL of 5 μM forward iTru5 primer, 2.5 μL of 5 μM reverse iTru7 primer, and 5 μL of product from the first PCR. The following were used as PCR conditions: initial denaturation at 95°C for 2 min; 10 cycles of 95°C for 20 s, 60°C for 15 s, and 72°C for 30 s; final extension at 72°C for 5 min. These PCR products were purified with Sera-Mag magnetic beads (Thermo Fisher Scientific, Waltham, MA, United States). We quantified the final products with a Qubit 2.0 Fluorometer (Thermo Fisher Scientific, Waltham, MA, United States) and pooled them in equal molar ratios for sequencing. Samples were sequenced using an Illumina MiSeq v2 600 cycle kit (Illumina, San Diego, CA, United States) at the Georgia Genomics and Bioinformatics Core (Athens, GA, United States).

### Metagenomic Libraries

Extracted DNA was sheared on a Bioruptor UCD-300 (Diagenode, Denville, NJ, United States) to an average size of about 500 bp. We input ∼100 ng of fragmented DNA into each reaction of a KAPA HyperPrep Kit (KAPA Biosystems, Wilmington, MA, United States) following manufacturer’s protocol at half volume reaction size with 14 PCR cycles using iTru adaptors and indexed primers (Glenn et al., 2019b). Samples were sequenced on an Illumina HiSeq 3000 with PE150 reads (Oklahoma Medical Research Foundation, Oklahoma City, OK, United States).

### 16S rRNA Bait Design

We used Prokka v1.11 with default settings, to annotate and extract all 16S rRNA sequences in GreenGenes v13.5 to ensure that only 16S rRNA regions were represented in the final bait set (Seemann, 2014). The GreenGenes database was chosen because it is freely available, widely used, and still reasonably comprehensive. Stretches of up to 25 Ns were replaced with T bases to facilitate probe design across short unknown regions. We then used USEARCH v8.1 (Edgar, 2010) to sort by length (large to short) and cluster (query coverage 90%, identity 90%) sequences, retaining one centroid from each cluster. We then designed 120-mer baits with flexible ∼50% overlap. These baits were then clustered using USEARCH (query coverage 75%, identity 78%), and one centroid per cluster retained. These clustering parameters were chosen because they allow for a comprehensive bait set, without an excessively large number of individual baits. Furthermore, hybridization baits can tolerate substantial sequence divergence, which we used to our advantage when collapsing at 78% identity (Li et al., 2013). This combination of bait design and bait length facilitates the bait set capturing 16S sequences not present in the GreenGenes database (both filling in gaps and reaching out to new, unknown, sequences).

### 16S rRNA Hybridization Capture Enrichments

Metagenomic libraries were combined into 500 ng pools of eight samples for rodents or two samples for mock communities. Target enrichments of each pool were performed using myBaits kit (Arbor Biosciences CAT # 308616, Ann Arbor, MI, United States) using the designed 16S rRNA Capture Baits following manufacturer’s protocol (v3.01) with a 24 h 65°C hybridization. Following hybridization, we used Dynabeads M-280 Streptavidin magnetic beads (Life Technologies, Carlsbad, CA, US) for capturing and washing each biotinyalted bait library. We then performed a post-enrichment amplification using Illumina P5/P7 primers (Illumina, San Diego, CA, United States) and KAPA HiFi HotStart reagents (KAPA Biosystems, Wilmington, MA, United States) using 98°C for 45 s, followed by 16–22 cycles of 98°C for 20 s, 60°C for 30 s, and 72°C for 60 s, ending with a final extension of 72°C for 5 min. PCR products were cleaned 1:1 with Sera-Mag beads (Glenn et al., 2019a), quantified on Qubit and pooled in equimolar ratios for sequencing paired-end 150 and 300 bp reads on Illumina HiSeq 3000 (Oklahoma Medical Research Foundation, Oklahoma City, OK, United States) and MiSeq (Georgia Genomics Bioinformatics Core, Athens, GA, United States), respectively.

### Simulating 16S rRNA Target Enrichment Data

To test the efficiency of our bait set under ideal conditions, we did an *in-silico* analysis to determine how well our baits works during an error- and bias- free hybridization process. Three metagenomes (i.e., Lindgreen synthetic metagenome (Lindgreen et al., 2016); Zymo Mock Community DS6306 genomes; and BEI Mock Community HM-276D) were used to simulate 16S rRNA capture data. In summary, a fasta file containing our 120-mer bait set was mapped to each metagenome fasta file (Supplementary Datas 1–3) using Burrows-Wheeler aligner (bwa) v.0.7.17 (Li and Durbin, 2009). Samtools v1.9 (Li et al., 2009) was used to convert the obtained sam file into a bam file. Following this, we obtained the mapping coordinates of the baits on the reference metagenomes and extracted the sequences + 200 bp to the upstream and downstream of the first position, if possible. Here, we sought to simulate a hybridization of the bait to the core of an ∼500 bp fragment while obtaining the flanking regions typically captured from use of biotinylated baits.

The software ART 2016.06.05 (Huang et al., 2012) was then used to simulate > 200,000 paired-end 150 bp fastq reads from these extended reference sequences from each metagenome. These fastq files were mapped to Greengenes 97% similarity database v.13.8 using BBmap v. 38.50 (Bushnell, 2014). For each metagenome, we recorded the number of paired reads mapped to Greengenes, number of forward reads, number of reverse reads and percentage average total mapped, and compared these results with those from real samples also mapped to the Greengenes database (see below) (Altschul et al., 1990).

### Sequencing Data Processing

**FIGURE 1:**
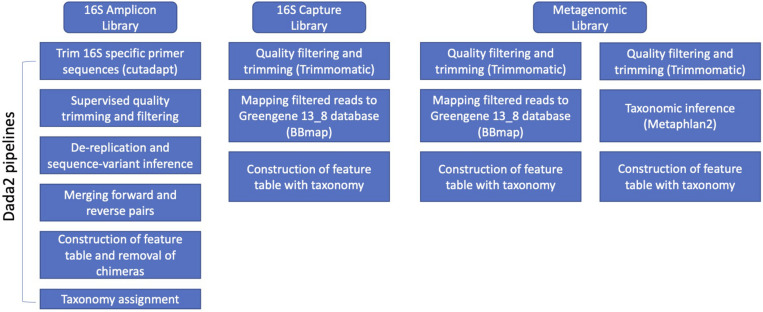
Overview of data analysis methods on the three library types (i.e., 16s amplicon, 16s hybridization bait capture, and metagenomic libraries).

After obtaining demultiplexed Illumina pair-end raw sequences, we used library specific pipelines to process the data (Figure 1). For 16S rRNA amplicon libraries, primers were removed using cutadapt v1.15 (Martin, 2011). Following this, DADA2 (v1.8) was used with customized parameters according to the quality profile of DNA sequences for quality trimming and filtering (truncLen was set to be 0.9 of sequencing length of forward reads and 0.8 for reverse reads; maxEE was set to be 6 for PE250 library, and 8 for PE300; maxN to be 0; default for the other parameters), de-replication and sequence-variant inference, merging paired-end reads, construction of feature tables, removal of chimeras, and taxonomy assignment (Callahan et al., 2016). The relative abundance was calculated by normalizing feature counts to the total counts of a sample. The taxonomy assignment was based on 97% clustered OTU of Greengenes v13.8 database using Naïve Bayesian Classifier as implemented in the DADA2 pipeline (Wang et al., 2007).

For 16S-cap libraries, the resulting quality filtered reads were mapped to the 97% clustered OTU based on Greengenes v13.8 database using BBmap v37.78 (Bushnell, 2014). The resulting mapping information was filtered, and a hit was recorded if both ends of paired read hit the same reference, or only one end of the paired read hit a reference. The relative abundance was calculated by normalizing feature counts to the total counts of a sample. Also, we assessed the presence of non-target reads in the quality-filtered dataset by (1) running MetaPhlAn2 v2.7.8 (Segata et al., 2012; Truong et al., 2015), and (2) mapping to the rat and mice genomes using Burrows-Wheeler aligner (bwa) v.0.7.17 (Li and Durbin, 2009).

For unenriched metagenomic libraries, Trimmomatic v0.36 (Bolger et al., 2014) was used for quality trimming using a sliding window of three nucleotides with an average Q > 20, and minimum length of 75 nucleotides. Reads that passed initial quality filtering (including both paired reads and orphan reads) were fed to MetaPhlAn2 v2.7.14 for taxonomy assignment (Segata et al., 2012; Truong et al., 2015). The relative abundance was estimated based on the database hit and marker gene length by MetaPhlAn2. To further compare to the results from 16S-cap analysis, we performed the same 16S mapping steps to the GreenGenes database as described for 16S-cap libraries for the unenriched libraries.

### Statistical Analysis

After obtaining feature tables from the above libraries using different bioinformatic tools, statistical summary and tests were carried out in R (R Development Core Team, 2010). Fold changes of observed relative abundance to theoretical relative abundance for the mock communities are calculated and ANOVA with Duncan’s multiple range test was used to compare different library types and analytical methods. Bray-Curtis distance matrix were generated using the relative abundance estimates from different libraries and methods as mentioned above, which was then analyzed by prinicle coordinate analysis (PCoA) to reveal clustering pattern. Additionally pairwise between-library/methods Bray-Curtis distance were compared by ANOVA with Duncan’s multiple range test. A significance level of 0.05 is used for the Duncan’s test.

## Results

### 16S rRNA Capture Bait Design

The 1,262,986 sequences comprising Greengenes v13.5 were annotated and 1,261,075 16S rRNA sequences were retained. A total of 117 sequences containing consecutive runs of 25 or more ambiguous bases (Ns) were removed. A total of 18,649 centroidal sequences were obtained from USEARCH clustering. From these sequences, 413,480 120-mer baits were designed. These baits were then clustered using USEARCH, retaining one centroid per cluster, for a total of 37,745 baits (i.e., unique probe sequences), indicating there are an average of ∼3,000 probes at each nucleotide position of the 16S rRNA.

### Sequencing Summary Statistics

A summary of average sequence statistics for each sample and library preparation type is given in Table 1. For the 16S rRNA amplicon data, the number of total raw read pairs per sample ranged from 49,828 for the Zymo mock community to 136,184 for the BEI mock community, with rodent fecal samples having intermediate depth. More reads (∼77%) remained from the rodent fecal samples after the denoising steps through the rigorous DADA2 pipelines vs. the mock communities. Low percentages of high quality reads remained following filtering for both the BEI and Zymo mock communities (38.7 and 48.8%, respectively). For the BEI mock community, initial index matching in R2 reads caused ∼30% loss of data (vs. less than 5% typically observed in other samples) and DADA2 quality trimming lost another ∼30% of data. For the Zymo mock community, the loss of data was mainly due to chimeric filtering (∼30% of data loss).

**TABLE 1:** A brief overview of the average summary statistics (i.e., number of samples, total raw read-pairs, average filtered/bar, average mapped/filtered) for each sample type of each library type (i.e., 16S amplicon libraries, 16S-cap enriched, and unenriched).

**Library type**	**Read length**	**Sample type**	**N samples**	**Total raw read-pairs**	**Total filtered reads**	**Average filtered/Raw (Mean ± SD)**	**Average mapped/Filtered (Mean ± SD)**
Amplicon-16S/V3V4	PE300	Rat feces	5	318,561	247,781	(77.3 ± 6.2)%	NA
Amplicon-16S/V3V4	PE300	BEI Mock	1	136,184	52,734	38.7%	NA
Amplicon-16S/V3V4	PE300	Zymo Mock	1	49,828	24,301	48.8%	NA
Amplicon-16S/V4	PE250	Mice feces	8	526,754	389,000	(77.6 ± 7.1)%	NA
Enriched	PE150	Mice feces	8	11,474,476	8,321,081	(70.1 ± 5.4)%	(59.1 ± 0.8)%
Enriched	PE150	Rat feces	5	9,470,428	6,450,541	(72.9 ± 2.1)%	(57.8 ± 4.1)%
Enriched	PE150	BEI Mock	1	8,203,396	5,345,638	76.7%	70.4%
Enriched	PE150	Zymo Mock	1	5,140,030	3,359,376	76.5%	70.1%
Enriched	PE300	Mice feces	8	1,573,122	1,050,608	(75.1 ± 3.2)%	(59.9 ± 2.1)%
Enriched	PE300	BEI Mock	1	1,108,481	737,309	75.2%	75.7%
Enriched	PE300	Zymo Mock	1	721,740	467,250	77.2%	73.8%
Unenriched	PE150	Mice feces	8	37,894,050	28,219,552	(68.6 ± 6.4)%	0.1%
Unenriched	PE150	Rat feces	5	28,448,468	16,266,683	(87.4 ± 0.9)%	0.1%
Unenriched	PE150	BEI Mock	1	8,889,636	6,263,379	71%	0.2%
Unenriched	PE150	Zymo Mock	1	7,001,503	4,985,957	70.2%	0.2%

For the unenriched libraries, the highest number of total raw read pairs ranged from 4,985,957 in the Zymo mock community to 28,219,552 in the insecticide-treated mouse feces. The percentage of reads retained after filtering was greater than 65% for all unenriched libraries. The average percentage of reads mapped to GreenGenes ranged from 0.1 to 0.2% in the BEI and Zymo mock communities.

For 16S-cap libraries, the PE150 reads had higher numbers of reads on average per sample type than PE300 reads. The highest number of raw reads (i.e., 11,474,476) was obtained for the insecticide-treated mouse feces with PE150 reads. The percentage of reads after filtering were greater than 70% for all 16S-cap libraries. The average percentage of mapped reads was greater than 50% for all 16S-cap libraries, with the highest percentage of mapping in the 16S-cap BEI mock community sequenced with PE300 at 75.7%. On average among all sample types, the proportion of on target reads was increased 435-fold when compared to unenriched libraries (range 283–499 fold increase, Suppplementary Table 2).

### 16S rRNA Target Enrichment Simulated Reads

Summary information for simulated reads is given in Table 2. We observed a higher percentage of total mapped reads in our simulated mock communities than for the real data from those communities (Table 2). For example, the real data from the Zymo mock community had an average total mapping of 78.15% to GreenGenes, compared to 91.43% from the simulated data. Similarly, the BEI mock community had an average total mapping of 78.62% for the real data, compared to 92.37% for the simulated data.

**TABLE 2:** Summary statistics for simulated data and real data from mock communities, libraries were enriched for 16S using the 16S-cap enrichment and sequenced on an Illumina MiSeq PE150 reads.

**Sample ID**	**Library type**	**Avg. no. of (Simulated) reads**	**No. of simulated reads**	**Matched pairs**	**Matched forward**	**Matched reverse**	**Total mapped**	**Percent of avg. total mapped**
**Simulated data**								
Zymo Mock	Enriched-PE150	412,520	206,260	171,708	190,964	186,216	377,180	91.43%
BEI Mock	Enriched-PE150	415,472	207,736	176,547	193,998	189,777	383,775	92.37%
Lindgreen et al. (2016)	Enriched-PE150	490,238	245,119	188,620	218,911	213,918	432,829	88.29%
**Real data**								
Zymo Mock	Enriched-PE150	3,904,480	1,952,240	1,314,654	1,548,323	1,503,225	3,051,548	78.15%
BEI Mock	Enriched-PE150	6,260,110	3,130,055	2,127,656	2,486,274	2,435,425	4,921,699	78.62%

### Validation on Mock Community Samples

**FIGURE 2:**
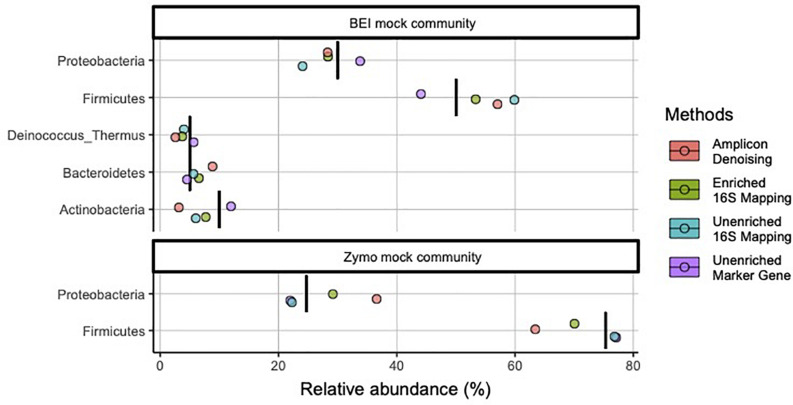
Relative abundance of bacterial phyla in mock community controls sequenced and analyzed using different methods. Phyla listed as components of the mock communities are shown. Black vertical bar in each row represents the nominal abundance of respective phylum. Row panel strips labels identify the mock communities; colors identify library type (i.e., amplicon, enriched 16S-cap, unenriched metagenomic library) and analyzing strategy (i.e., denoising, 16Smapping, and marker gene).

We initially prepared amplicon libraries, unenriched metagenomic libraries, and performed target enrichment for 16S rRNA (i.e., 16S-cap) on metagenomic libraries using two mock communities (Table 1). At the phylum level both samples appear to provide accurate identification of the microbes with good estimates of abundance, regardless of library type or data analysis method used (Figure 2). Additionally, in both the unenriched and 16S-cap libraries analyzed with a 16S mapping approach, Cyanobacteria was found in low abundance even though it was not expected to be present in the mock community. However, when analyzing the unenriched library using marker gene approach, Cyanobacteria was not found and instead Ascomycota was identified.

**FIGURE 3:**
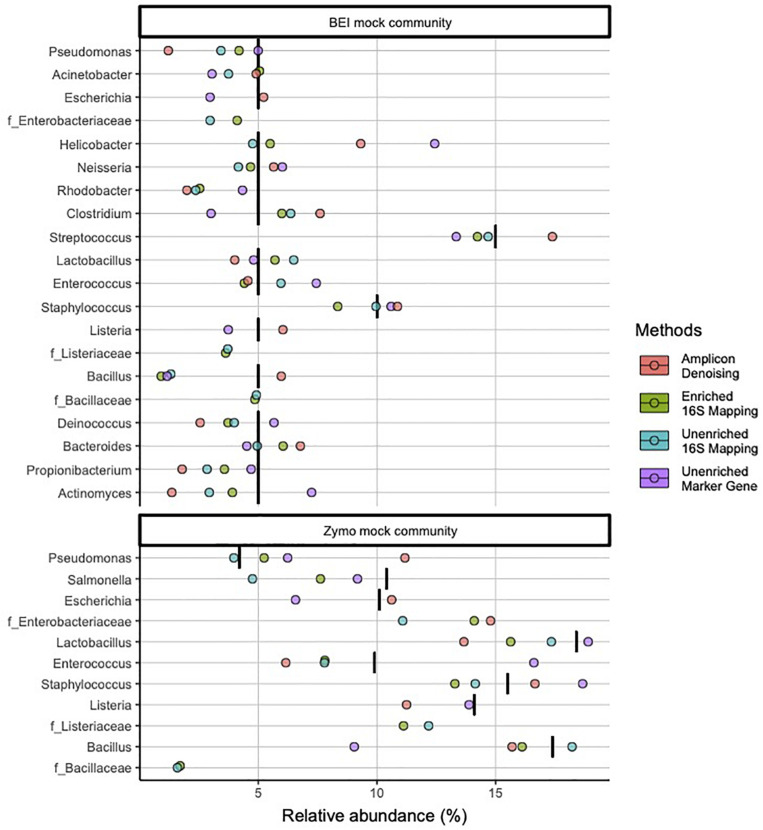
Relative abundance of bacterial genera in mock community controls sequenced and analyzed using different methods. Genera listed as components of the mock communities are shown. Three families with no genus identification, *Enterobacteriaceae*, *Listeriaceae*, *Bacillaceae*, are plotted below the probable genus (*Escherichia, Listeria*, and *Bacillus*), respectively. Black vertical bar in each row represents the nominal abundance of respective genus. Row panel strips labels identify the mock communities; color identify library type (i.e., amplicon, enriched 16S-cap, unriched metagenomic library) and analyzing strategy (i.e., denoising, 16Smapping, and marker gene).

At the genus level, 16S-cap and unenriched libraries reflect more accurate microbial community composition and abundance for most taxa (Figure 3). The 16S-cap and unenriched libraries with 16S mapping missed three genera: *Escherichia*, *Listeria*, and *Bacillus* for both mock community samples. However, three families with no genus identification, *Enterobacteriaceae*, *Listeriaceae*, *Bacillaceae*, were found, suggesting these are likely the missing genera, and are represented at a family level. In comparison, 16S rRNA amplicon-based analysis identified nearly all genera in mock samples, however, its estimates of abundance for *Actinomyces*, *Propionibacterium*, *Pseudomonas*, and *Rhodobacter* all greatly deviate from the nominal compositions. The unenriched metagenomic libraries analyzed with a marker-gene approach were able to identify all 18 genera in the mock communities, however, its estimate of *Bacillus* abundance in both mock communities deviate from the nominal composition (Figure 3).

**FIGURE 4:**
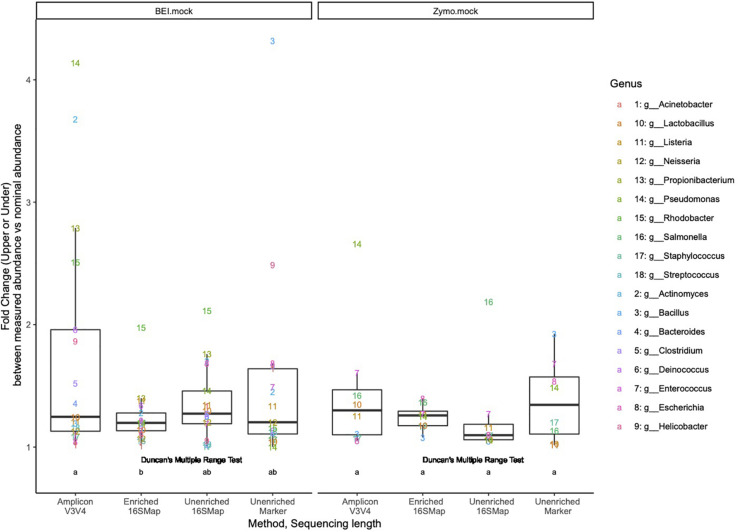
Fold change (i.e., upper or under) comparing the relative abundances of respective genera in each library to its nominal abundance. Duncan’s multiple range test was performed to compare each library type for each mock community. Letters indicate whether significant differences were detected.

In the BEI mock community libraries, relative abundance estimates in the 16S-cap libraries were more accurate than the amplicon and unenriched libraries as measured by fold change being very close to 1 (Figure 4). In the amplicon library, several genera (i.e., *Pseudomonas*, *Actinomyces*, *Propionilbacterium*, and *Rhodobacter*) are beyond the twofold change of their nominal compositions. In particular one genus, *Rhodobacter*, proved to be challenging for all three library preparation methods for accurate estimation of relative abundance. Duncan’s multiple range test revealed that there were significant differences (*p* 0.05) between the BEI mock community amplicon and 16S-cap libraries, whereas the unenriched libraries were not found to be significantly different than the amplicon or 16S-cap libraries. For the Zymo mock community libraries, relative abundance estimates in the 16S-cap libraries are more accurate than relative abundance estimates for the amplicon library. However, Duncan’s multiple range test did not detect a significant difference between the three library types (i.e., amplicon, unenriched, and enriched) (Figure 4).

### Validation on Fecal Samples

**FIGURE 5:**
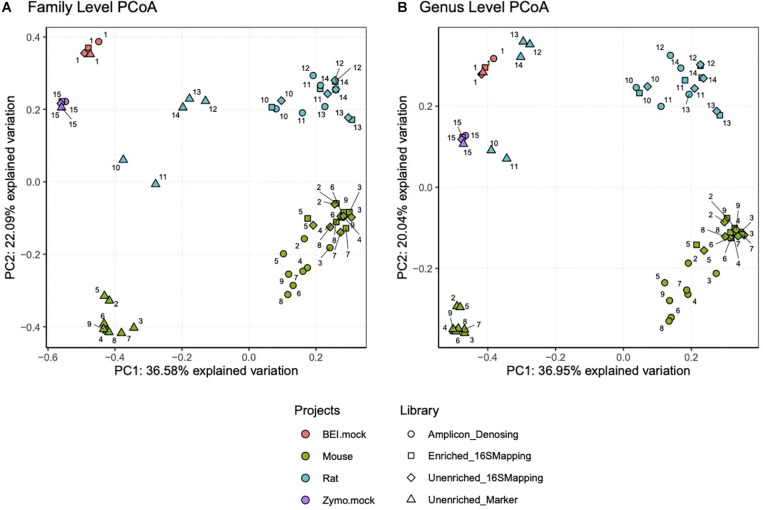
PCoA plots were constructed using Bray-Curtis dissimilarity matrix at a family level **(A)** and genus level **(B)**. Each project is represented by a colored dot (i.e., orange = BEI mock community, green = mouse samples, blue = rat samples, and purple = Zymo mock community). Each library type, sequencing read length and data analysis method is represented by a different shape (i.e., circle = amplicon library, square = 16S-cap enriched PE150 reads, diamond = unenriched PE150 analyzed with 16S mapping and triangle = unenriched PE150 analyzed with metagenome mapping). Numbers represent sample number.

Principle coordinate analysis was performed on mock community samples and additional samples from laboratory mice and rats to further validate the 16S-cap method. When Bray-Curtis was used to construct the dissimilarity matrix, which considers abundance estimates, we found that regardless of analyses at the level of family (Figure 5A, left) or genus (Figure 5B, right) similar themes emerged. We observed that the mock community samples were similar to each other regardless of library type. Conversely, in the mouse and rat samples, we found that the unenriched libraries analyzed with a marker-gene approach grouped together separately from amplicon, unenriched, and 16S-cap libraries, all of which were analyzed with the 16S mapping approach.

**FIGURE 6:**
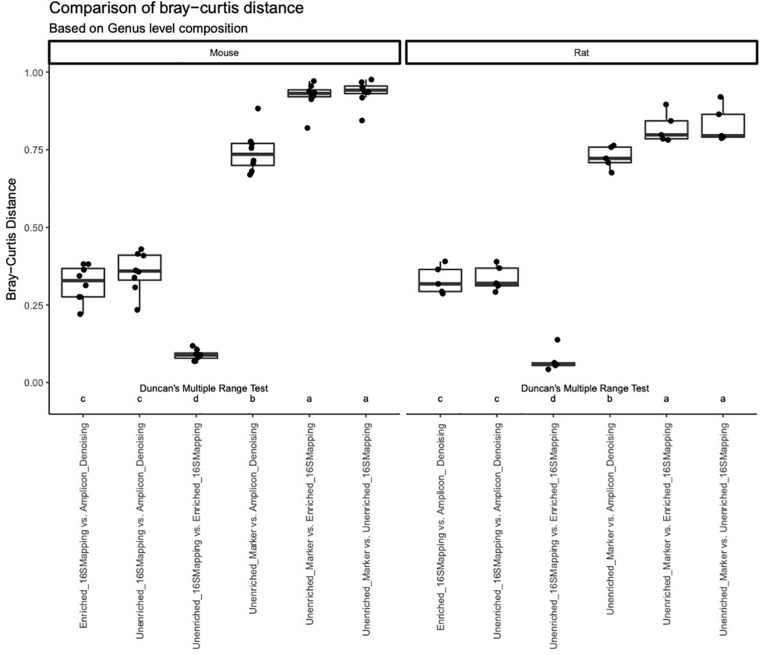
A comparison of the Bray-Curtis distance metric was performed for each library type at a genus level using box plots. Bray-Curtis distance is indicated on the *y*-axis. Library type is indicated on the *x*-axis. Duncan’s multiple range test was performed to compare each library type for each mock community. Letters indicate whether significant differences were detected.

A comparison of Bray-Curtis distance was performed for rodent fecal samples at the level of family and genus (Figure 6). This analysis revealed similar trends regardless of sample type or taxonomic rank. The 16S-cap and unenriched libraries analyzed with 16S mapping approach showed to be the most similar to each other, with a dissimilarity rate below 0.25. Bray-Curtis dissimilarity was higher when comparing the amplicon libraries to both 16S-cap and unenriched libraries. When comparing the unenriched libraries analyzed with two different analysis strategies (i.e., mapping reads to GreenGenes vs. gene-marker approach), we observed the highest degree of dissimilarity at both the family and genus levels with dissimilarity rates at approximately 0.75. *Post-hoc* analysis revealed that there were significant differences when comparing the unenriched and 16S-cap libraries to all other library types, regardless of sample type or taxonomic rank (Figure 6).

## Discussion

Given the limitations of 16S rRNA amplicon and shotgun metagenomic libraries outlined in the introduction, we sought to provide an alternative method to identify microbial community composition by creating a 16S rRNA hybridization capture assay (i.e., 16S-cap). Our study revealed two important things: (1) our 16S-cap method is an efficient way to obtain sequences from the complete 16S rRNA gene to accurately reflect microbial community composition and abundance and (2) bioinformatic analysis methods greatly influence community composition in host-related samples, regardless of library type. In our study we observed that sequences from 16S-cap were not significantly different than sequences from unenriched shotgun libraries when analyzed using similar bioinformatic methods and databases. However, we did find that the 16S-cap assay requires far fewer reads, thus allowing enriched libraries to be characterized on benchtop sequencers, including Illumina MiSeq instruments, at reasonable cost while overcoming the previously mentioned limitations with direct 16S rRNA approaches and metagenomic approaches. These limitations include selection and drift bias in PCR during amplicon library preparation and the potential for non-target DNA (e.g., human DNA) in metagenomic libraries, which can lead to errors in downstream analyses.

Enrichment for genes of interest is an important technique in characterizing complex environmental and host-related samples. Previous studies have found other capture enrichment methods to increase the proportion of on target reads from ∼0.1% in unenriched shotgun libraries to ∼60% in enriched libraries (Gasc and Peyret, 2018). Similarly, we found 0.1–0.2% of unenriched libraries to map to the16S rRNA, whereas 58–76% of the enriched reads mapped to the 16S rRNA (Table 1). On average we achieved a 435-fold increase in reads mapped to the 16S rRNA in our 16S-cap libraries compared to the unenriched libraries (Supplementary Table 2). *In silico* simulations of 16S-cap revealed that under ideal conditions, 88–92% mapping to the 16S rRNA from mock communities could be achieved. Therefore, our 16S-cap enrichment process helps to achieve a very high percentage of on-target reads, but not quite as high as theoretically possible.

Our 16S-cap method identified several species that were not expected in the theoretical targets of the mock communities, which may be attributed to several factors. First, the lack of genus identification may be due to the mapping methods or clustering level used in data analysis rather than the library preparation method. Both the 16S-cap and unenriched libraries analyzed with a 16S mapping method failed to identify three genera *Escherichia*, *Listeria*, and some *Bacillus* in the mock communities. However, there are three familes, *Enterobacteriaceae*, *Listeriaceae*, and *Bacillaceae*, are associated with our missing genera. Thus, it appears that reads for these three genera appear to be present, but are not being assigned appropriately by the bioinformatic program at the genus level. By designating these unidentified genera as *Escherichia*, *Listeria*, and *Bacillus* respectively, the 16S-cap library is highly accurate in terms of taxonomic classification and abundance. Taxonomic misassignment is a known problem with 16S mapping methods (Park and Won, 2018; Abellan-Schneyder et al., 2021), and new software is in development (Schloss and Westcott, 2011; Pollock et al., 2018; Zinger et al., 2019; Djemiel et al., 2020). Moreover, several other studies have found bioinformatic databases have difficulty assigning *Escherichia*, *Listeria*, and *Bacillus* at a genus level (Park and Won, 2018; Abellan-Schneyder et al., 2021). Additional work on the mapping and assignment processes used here, as well as comparisons of newly developed and commonly used bioinformatic software is beyond the scope of this paper, but warranted in future work.

We compared theoretical target values of the BEI resources and Zymo mock communities to all three library types (i.e., amplicon, unenriched, and 16S-cap) (Figures 3, 4). We find that the 16S-cap libraries are representative of the target abundance values of the mock communities (Figure 3). *Post-hoc* analysis revealed that the 16S rRNA amplicon library and 16S-cap library made from the BEI mock community were significantly different from each other (*p* ≤ 0.05) based on relative abundance. A PCoA revealed that in the mouse and rat samples the unenriched libraries analyzed with a marker-gene approach grouped together separately from 16S rRNA amplicon libraries and 16S-cap and unenriched libraries analyzed with taxonomic binning approach (Figure 5). Thus, enrichment and amplicon sequencing result in similar library composition, as do 16S-cap and unenriched libraries analyzed with a 16S taxonomic binning approach. This indicates that our 16S-cap method may be less biased than 16S amplification, but that analysis methods or the reference database may greatly influence community composition results. Walsh et al. (2018) analyzed different species classifiers using marker gene approaches and taxonomic binning, and found that the results of the marker gene approach (i.e., MetaPhlAn2) were different from taxonomic binning methods. Taxonomic binning methods are influenced by the size of the reference genome, whereas marker gene approaches are not (Droge and McHardy, 2012; Balvociute and Huson, 2017; Walsh et al., 2018). The use of hybridization capture baits may help alleviate some of these issues.

Other groups have designed a more limited bait set to hybridize all known 16S rRNA gene sequences by focusing on highly conserved regions and incorporating ambiguities (Gasc and Peyret, 2018). When validating their bait set on a mock community, they found that they detected 24 of 26 genera tested, and that two less abundant species (i.e., *Methanobrevibacter smithii* and *Methanococcus aelocius* at 0.00006%) were missed. In addition, Cariou et al. (2018) tested hybridization capture probes designed by Gasc and Peyret (2018) on a previously characterized pea aphid and found their enriched libraries to be representative of the bacterial population. There are some key differences between the design of our baits set and Gasc and Peyret (2018). Foremost, is the number of baits included in the bait set. Our bait set included 37,745 120-mer baits and was designed from all 16S rRNA gene sequences in GreenGenes, whereas Gasc and Peyret bait set include 15 baits that are 28—50-mer and was designed by focusing on highly conserved regions of the 16S rRNA and incorporation of degenerate sites. We used 120-mer baits because 120 nt is the maximum practical size for the Arbor Biosciences platform and it maximizes the tolerance of non-complementary bases with reasonable hybridization times. Additionally, using more baits with more sequence variation among the baits helps to capture a greater range of diverse targets and thus generates more accurate abundance estimates of the full range of community members. Having a more extensive bait set, such as ours, may reduce some of the previous challenges, demonstrated by the ability to detect all genera in the mock communities. These aspects are critical when studying environmental and host-related samples and searching for rare taxa. In addition, the use of longer hybridization times or “double capture” (i.e., when captured product is captured again) can improve the percentage of on target reads and help capture rare sequences. Future work to identify the optimal bait set(s) for various microbial communities and research objectives should include a direct comparison of the Gasc and Peyret (2018) bait set verses our bait set.

Preparing 16S-cap libraries can most readily be accomplished by using an existing enrichment kit, which ranges in cost from $1,500 to $5,200 depending on the number of reactions purchased. To reduce reagent costs and hands-on time, we have successfully pooled multiple samples (see section “16S rRNA Hybridization Capture Enrichments”), which is commonly done (Glenn and Faircloth, 2016). For example, pooling samples in groups of eight reduces capture costs from $93.75 per sample to $11.72 per sample (Supplementary Table 3). Larger numbers of samples can be pooled to further reduce costs, but there are tradeoffs (see Glenn and Faircloth, 2016). Our baitset is commercially available from Arbor Biosciences in ready-to-use kit format, and the bait sequences are freely available to the scientific community (Supplementary Data 4). Thus, our baits can be modified and/or synthesized by any strategy any researcher desires.

Sequencing 16S-cap libraries require less extensive sequencing than unenriched shotgun metagenomic libraries, which reduces costs (Supplementary Tables 4, 5). It is important to note that the number of reads obtained for 16S-cap libraries here (Table 1) is far more than are necessary or would be reasonable when implementing this strategy. For example, a 100-fold 16S-cap enrichment sequenced on an Illumina MiSeq Nano PE150 provides a cost-savings of approximately $315 compared to an unenriched metagenomic shotgun library requiring 1 million reads (Supplementary Table 4). Indeed, 16S-cap makes it economically and logistically reasonable to routinely screen for 16S segments from enriched shotgun metagenomic libraries on Illumina MiSeqs. 16S-cap decreases costs when using a production scale Illumina sequencer (e.g., Illumina NovaSeq) to less than $0.10 per sample when achieving a 100-fold enrichment (Supplementary Table 5). However, because production scale sequencers produce 400–2,500 million read pairs, to achieve low cost for samples needing relatively few reads, each run requires huge numbers of samples or a mixture of some samples needing large numbers of reads (i.e., a mixture of projects; see Glenn et al., 2019a). Due to the limited savings possible on production sequencing costs (Supplementary Table 4), the savings in data transfer, storage, and compute time may be more significant than savings in sequencing costs.

In summary, our data demonstrates that the 16S-cap assay and unenriched shotgun metagenomic libraries produce very similar community profiles. Importantly, our 16S-cap library is produced from a metagenomic library, which eliminates primer (though not all PCR) biases. Additionally, our 16S-cap assay provides a deeper community profile (i.e., more 16S reads that can be queried to a database) with far fewer reads than the unenriched shotgun metagenomic libraries. In host-related samples, we routinely achieved > 400-fold enrichment. Thus, expensive deep sequencing is unnecessary for 16S-cap libraries because a few thousand reads provide the same number of 16S rRNA sequences as millions of shotgun reads. By trading modest additional library preparation costs for reduced sequencing costs (Supplementary Tables 3–5), 16S cap is economical and opens up the possibility of adding deep taxonomic sampling to studies that are capturing other genes of interests e.g., antibiotic resistance genes (Guitor et al., 2019; Oladeinde et al., 2019; Thomas et al., 2020). In comparison to amplicon libraries, the 16S-cap assay will be more expensive, however, it provides superior microbial community resolution, increased accuracy of relative abundance, an orthoganol approach to marker genes, and greater flexibility in terms of sequencer and kit choice. We believe that our bait set is a valuable tool to efficiently and accurately identify microbial community composition and would be well-suited to be used in combination with other bait sets targeting different genes of interest (e.g., antimicrobial resistance baits).

## Data Availability Statement

The datasets presented in this study can be found in online repositories. The names of the repository/repositories and accession number(s) can be found below: https://www.ncbi.nlm.nih.gov/sra/PRJNA689808.

## Author Contributions

TG conceived of the project. JW, JT, TK, BG, KL, and TG designed the experiments. JW, TK, and BG performed the experiments. AD and BB designed the baits. JW and NB-V analyzed the data. AD, BB, KL, OR, J-SW, and TG provided funding and resources. MB wrote the manuscript. JW, TK, and NB-V wrote sections of the manuscript. MB and JW produced figures and tables. All authors critically reviewed, edited, and approve of this work.

## Disclaimer

This report was prepared as an account of work sponsored by agencies of the United States Government. Neither the United States Government, nor any agency thereof, nor any of their employees makes any warranty, express or implied, or assumes any legal liability or responsibility for the accuracy, completeness, or usefulness of any information, apparatus, product, or process disclosed or represents that its use would not infringe privately owned rights. Reference herein to any specific commercial product, process, or service by trade name, trademark, manufacturer, or otherwise does not necessarily constitute or imply its endorsement, recommendation, or favoring by the United States Government or any agency thereof. The views and opinions of authors expressed herein do not necessarily state or reflect those of the United States Government or any agency thereof.

## Conflict of Interest

The EHS DNA lab provides oligonucleotide aliquots and library preparation services at cost, including some oligonucleotides and services used in this manuscript (baddna.uga.edu). BB and AD were employed by, and thereby have financial interest in, Daicel Arbor Biosciences, who provided the in-solution capture reagents used in this work. The remaining authors declare that the research was conducted in the absence of any commercial or financial relationships that could be construed as a potential conflict of interest.
