# Supplementary material for: Improved Microbial Community Characterization of 16S rRNA via Metagenome Hybridization Capture Enrichment
Source: Front Microbiol. 2021 Apr 27;12:644662. doi: 10.3389/fmicb.2021.644662 (PMC8110821; doi:10.3389/fmicb.2021.644662)
Supplement: Supplementary file 1 [file Data_Sheet_1.PDF]

# *Supplementary Material*

## 1 Supplementary Data

**Supplementary Data 1.** Lindgreen genome FASTA file used for *in silico* analysis of simulating 16S rRNA capture data (Lindgreen, Adair, & Gardner, 2016) 3.95 GB].

**Supplementary Data 2.** Zymo mock community FASTA file used for *in silico* analysis of simulating 16S rRNA capture data, 72.4 MB [https://www.dropbox.com/s/neu04baa5wili25/Supplemental_Data-02_BEI_genomes.fasta?dl=0].

**Supplementary Data 3.** BEI mock community FASTA file used for *in silico* analysis of simulating 16S rRNA capture data, 63 MB [https://www.dropbox.com/s/5196mzctbwjz7cx/Supplemental_Data-03_Zymo_genomes.fasta?dl=0].

**Supplementary Data 4.** 16S rRNA bait capture sequences [https://www.dropbox.com/s/pubx5n8r4syfjgt/Supplemental_Data-04_16S_Baits.fas?dl=0].

## 2 Supplementary Figures and Tables

### 2.1 Supplementary Figures

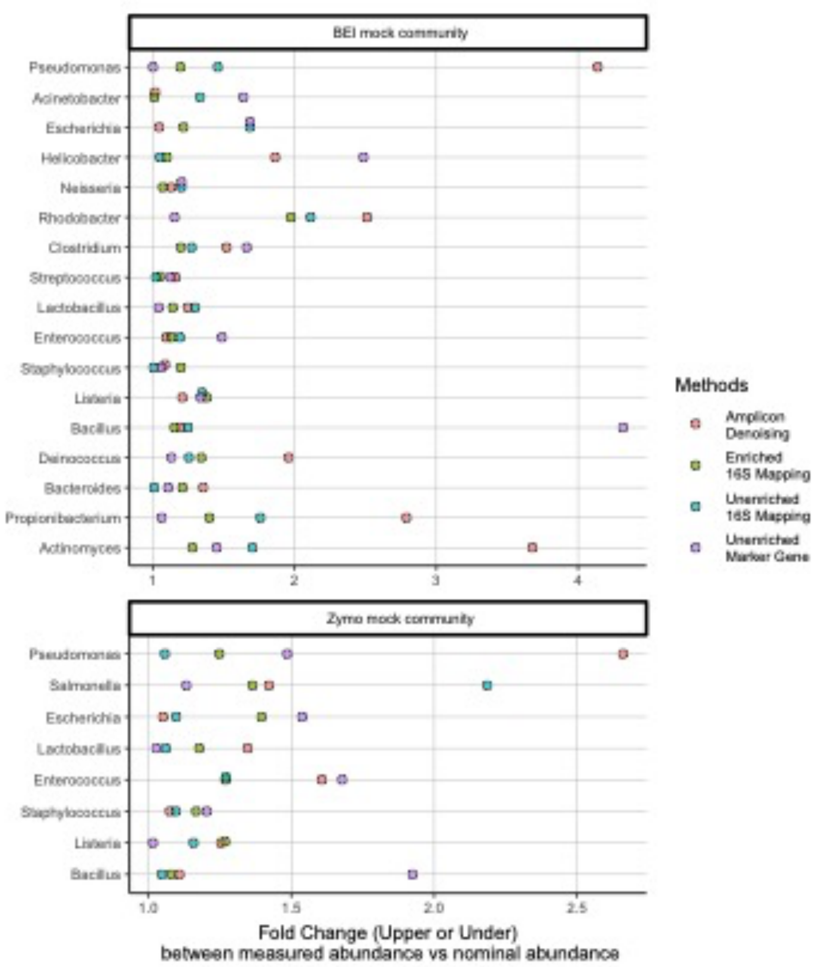


**Supplementary Figure 1.** Fold change (i.e., upper or under) comparing the relative abundances of respective genera in each library to its nominal abundance. Row panel strips labels identify the mock communities; colors identify library type (i.e., amplicon, enriched 16S-cap, unenriched metagenomic library) and analyzing strategy (i.e., denoising, 16Smapping, and marker gene).

## 2.2 Supplementary Tables

**Supplementary Table 1.** Nominal composition of the three mock communities (i.e., BEI HM-276D, Zymo D6306, and ATCC-MSA 1002) used in this study.

| Name | BEI | Zymo | ATCC | Phylum | Class | Order | Family |
|---|---|---|---|---|---|---|---|
| Deinococcus radiodurnas | 5% | | 5% | Deinocccus_Therumus | Deinococci | Deinococcales | Deinococcaceae |
| Actinomyces odontolyticus | 5% | | | Actinobacteria | Acintobacteria | Actinomycetales | Actinomycetaceae |
| Propionibacterium acnes | 5% | | | Actinobacteria | Acintobacteria | Actinomycetales | Propionibacteriaceae |
| Bifidobacterium adoscentis | | | 5% | Actinobacteria | Acintobacteria | Bifidobacteriales | Bifodbacteriaceae |
| Bacteroides vulgatus | 5% | | 5% | Bacteroidetes | Bacteroidia | Bacteroidales | Bacteroidaceae |
| Porphyomonas gigivalis | | | 5% | Bacteroidetes | Bacteroidia | Bacteroidales | Porphyromonadaceae |
| Bacillus cereus | 5% | | 5% | Firmicutes | Bacilli | Bacillales | Bacillaceae |
| Bacillus subtilis | | 17.4% | | Firmicutes | Bacilli | Bacillales | Bacillaceae |
| Listeria monocytogenes | 5% | 14.1% | | Firmicutes | Bacilli | Bacillales | Listeraiceae |

| | | | | | | | |
|---|---|---|---|---|---|---|---|
| Staphylococcus aureus | 5% | 15.5% | 5% | Firmicutes | Bacilli | Bacillales | Staphylocccaceae |
| Staphylococcus epidermidis | 5% | | 5% | Firmicutes | Bacilli | Bacillales | Staphylocccaceae |
| Enterococcus faecalis | 5% | 9.9% | 5% | Firmicutes | Bacilli | Lactobacillales | Enterococcaceae |
| Lactobacillus fermentum | | 18.4% | | Firmicutes | Bacilli | Lactobacillales | Lactobacillaceae |
| Lactobacillus gasseri | 5% | | 5% | Firmicutes | Bacilli | Lactobacillales | Lactobacillaceae |
| Streptococcus agalactiae | 5% | | 5% | Firmicutes | Bacilli | Lactobacillales | Streptococcaceae |
| Streptococcus mutans | 5% | | 5% | Firmicutes | Bacilli | Lactobacillales | Streptococcaceae |
| Streptococcus pneumoniae | 5% | | | Firmicutes | Bacilli | Lactobacillales | Streptococcaceae |
| Clostridium beijerinckii | 5% | | 5% | Firmicutes | Clostridia | Clostridiales | Clostridiaceae |
| Rhodobacter sphaeroides | 5% | | 5% | Proteobacteria | Alphaproteobacteria | Rhodobacterales | Rhodobacteraceae |
| Neisseria meningitidis | 5% | | 5% | Proteobacteria | Betaproteobacteria | Neisseriales | Neisseriaceae |
| Helicobacter pylori | 5% | | 5% | Proteobacteria | Epsilonproteobacteria | Campylobacterales | Helicobacteraceae |
| Escherichia coli | 5% | 10.1% | 5% | Proteobacteria | Gammaproterobacteria | Enterobacteriales | Enterobacteriaceae |

| | | | | | | | |
|---|---|---|---|---|---|---|---|
| Salmonella enterica | | 10.4% | | Proteobacteria | Gammaproterobacteria | Enterobacteriales | Enterobacteriaceae |
| Acinetobacter baumannii | 5% | | 5% | Proteobacteria | Gammaproterobacteria | Pseudomonadales | Moraxellaceae |
| Pseudomonas aeruginosa | 5% | 4.2% | 5% | Proteobacteria | Gammaproterobacteria | Pseudomonadales | Pseudomonadaceae |
| Cutibacterium acnes | | | 5% | Actinobacteria | Actinobacteria | Actinomycetales | Propionibacteriaceae |
| Schaalia odontytica | | | 5% | Actinobacteria | Actinobacteria | Actinomycetales | Actinomycetaceae |

**Supplementary Table 2.** Fold enrichment of 16S-cap libraries compared to unenriched libraries for each sample type, average fold change by sample type and average fold change of 16S-cap libraries from all samples.

| Sample Type | Unenriched No. of High Quality Reads | Unenriched PE150 Total Mapped | Unenriched Percent Mapped | Enriched No. of High Quality Reads | Enriched-PE150 Total Mapped | Enriched Percent Mapped | Fold Change | Average Fold Change by Sample Type |
|---|---|---|---|---|---|---|---|---|
| Mice feces | 3,779,639 | 5400 | 0.142870787 | 1,194,067 | 700650 | 58.67761189 | 409.70 | 461.87 |
| Mice feces | 3,635,343 | 4295 | 0.118145661 | 983,572 | 571761 | 58.13107734 | 491.03 | |
| Mice feces | 7,279,499 | 10227 | 0.140490438 | 2,090,890 | 1256196 | 60.07948768 | 426.64 | |
| Mice feces | 7,635,458 | 10297 | 0.13485766 | 2,310,664 | 1355089 | 58.64500421 | 433.87 | |
| Mice feces | 3,336,870 | 3910 | 0.117175677 | 985,870 | 577908 | 58.61908771 | 499.27 | |
| Mice feces | 4,044,884 | 4824 | 0.119261764 | 1,200,140 | 704760 | 58.72314897 | 491.39 | |
| Mice feces | 3,769,616 | 4967 | 0.131764084 | 1,314,682 | 795110 | 60.47926419 | 458.00 | |
| Mice feces | 4,412,741 | 5381 | 0.121942348 | 1,394,591 | 826616 | 59.27300549 | 485.07 | |
| Control rat feces | 4,514,857 | 10033 | 0.222221878 | 2,116,491 | 1337220 | 63.18099156 | 283.31 | 427.15 |
| Control rat feces | 6,959,148 | 8240 | 0.118405299 | 873,582 | 473766 | 54.23257347 | 457.02 | |
| Rat feces | 7,225,709 | 9823 | 0.135945137 | 1,760,254 | 1046702 | 59.46312294 | 436.41 | |
| Rat feces | 4,230,171 | 4772 | 0.112808678 | 2,358,268 | 1250761 | 53.03727142 | 469.15 | |
| Rat feces | 5,518,583 | 6624 | 0.120030812 | 2,361,833 | 1391474 | 58.91500373 | 489.83 | |
| Zymo Mock Community | 8,889,636 | 18282 | 0.205655215 | 5,140,030 | 3601840 | 70.07429918 | 339.74 | 349.92 |
| BEI Mock Community | 7,001,503 | 13659 | 0.195086684 | 8,203,396 | 5778977 | 70.44615425 | 360.10 | |
| | | | | | | | Avg. Fold Change | 435.37 |

**Supplementary Table 3.** The cost of 16S bait enrichment per capture and per sample for 16S-cap based on list prices at https://arborbiosci.com/genomics/targeted-sequencing/mybaits/mybaits-expert/mybaits-expert-16s-hyb/.

| captures | reagent cost $ | $/capture | number of samples (libraries) pooled per capture (Costs) 8 | 12 | 24 | 48 | 96 |
|---|---|---|---|---|---|---|---|
| 16 | $1,500.00 | $93.75 | $11.72 | $7.81 | $3.91 | $1.95 | $0.98 |
| 48 | $3,400.00 | $70.83 | $8.85 | $5.90 | $2.95 | $1.48 | $0.74 |
| 96 | $5,200.00 | $54.17 | $6.77 | $4.51 | $2.26 | $1.13 | $0.56 |

**Supplementary Table 4.** The cost of sequencing reads on two exemplar Illumina sequencers (i.e., Illumina MiSeq and Illumina NovaSeq) with a few different available kits, and the fold-change enrichment with calculated cost relative to 1 million shotgun reads. Prices were current as of October 2020.

| | | | | | fold-change enrichment (with calculated cost relative to 1M shotgun reads) | | | | | | |
|---|---|---|---|---|---|---|---|---|---|---|---|
| Sequencer | Kit | Reagent cost | Millions of read pairs | $/M read pairs | 100 | 200 | 300 | 400 | 500 | 600 | 700 |
| MiSeq | Nano PE150 | $319.00 | 1 | $319.00 | $3.19 | $1.60 | $1.06 | $0.80 | $0.64 | $0.53 | $0.46 |
| | Nano PE250 | $381.00 | 1 | $381.00 | $3.81 | $1.91 | $1.27 | $0.95 | $0.76 | $0.64 | $0.54 |
| | Micro PE150 | $479.00 | 4 | $119.75 | $1.20 | $0.60 | $0.40 | $0.30 | $0.24 | $0.20 | $0.17 |
| | v3 PE300 | $1540.08 | 25 | $61.60 | $0.62 | $0.31 | $0.21 | $0.15 | $0.12 | $0.10 | $0.09 |
| NovaSeq | SP PE150 | $1,650.00 | 400 | $4.13 | $0.04 | $0.02 | $0.01 | $0.01 | $0.01 | $0.01 | $0.01 |
| | SP PE250 | $2,250.00 | 400 | $5.63 | $0.06 | $0.03 | $0.02 | $0.01 | $0.01 | $0.01 | $0.01 |
| | S4 PE150 XP | $3,750.00 | 2500 | $1.50 | $0.02 | $0.01 | $0.01 | $0.00 | $0.00 | $0.00 | $0.00 |

**Supplementary Table 5.** The cost of sequencing reads on two exemplar Illumina sequencers (i.e., Illumina MiSeq and Illumina NovaSeq) with a few different available kits, and the fold-change enrichment with calculated savings relative to 1 million shotgun reads. Prices were current as of October 2020.

| | | | | | fold-change enrichment (with calculated cost Savings relative to 1M shotgun reads) | | | | | | |
|---|---|---|---|---|---|---|---|---|---|---|---|
| Sequencer | Kit | Reagent cost | Millions of read pairs | $/M read pairs | 100 | 200 | 300 | 400 | 500 | 600 | 700 |
| MiSeq | Nano PE150 | $319.00 | 1 | $319.00 | $315.81 | $317.41 | $317.94 | $318.20 | $318.36 | $318.47 | $318.54 |
| | Nano PE250 | $381.00 | 1 | $381.00 | $377.19 | $379.10 | $379.73 | $380.05 | $380.24 | $380.37 | $380.46 |
| | Micro PE150 | $479.00 | 4 | $119.75 | $118.55 | $119.15 | $119.35 | $119.45 | $119.51 | $119.55 | $119.58 |
| | v3 PE300 | $154.08 | 25 | $61.60 | $60.99 | $61.30 | $61.40 | $61.45 | $61.48 | $61.50 | $61.52 |
| NovaSeq | SP PE150 | $1,650.00 | 400 | $4.13 | $4.08 | $4.10 | $4.11 | $4.11 | $4.12 | $4.12 | $4.12 |
| | SP PE250 | $2,250.00 | 400 | $5.63 | $5.57 | $5.60 | $5.61 | $5.61 | $5.61 | $5.62 | $5.62 |
| | S4 PE150 XP | $3,750.00 | 2500 | $1.50 | $1.49 | $1.49 | $1.50 | $1.50 | $1.50 | $1.50 | $1.50 |
